# The Role of Task-Specific Response Strategies in Blocked-Cyclic Naming

**DOI:** 10.3389/fpsyg.2016.01955

**Published:** 2017-01-04

**Authors:** Eva Belke

**Affiliations:** Sprachwissenschaftliches Institut, Ruhr-Universität BochumBochum, Germany

**Keywords:** lexical retrieval, language production, semantic interference

## Abstract

In word retrieval, speakers need to select a lexical entry among several co-activated candidates for lexicalization. How a target entry is selected is a matter of ongoing debate. Semantic context effects on naming times, as seen in the blocked-cyclic naming paradigm, are of specific interest to this debate. In the standard version of this paradigm, participants name lists of objects compiled from several repetitions (cycles) of a small set of semantically related objects (homogeneous context) or unrelated objects (heterogeneous context). In the first cycle, participants typically show either no context effect or semantic facilitation. From cycle two onward, they display a stable semantic interference effect that does not increase over cycles. In this review, I demonstrate that the early semantic facilitation effect is only observed consistently in studies that present homogeneous and heterogeneous lists in a blocked fashion. With this design, participants can easily pick up on the categorical relatedness of the items in semantically related contexts and apply this knowledge strategically. In principle, such response strategies can be easily tied in with existing models of lexical selection, but they are incompatible with accounts of semantic context effects that take the semantic facilitation effect in cycle 1 to be a consequence of processes inherent to the lexicalization process. Users of the blocked-cyclic naming paradigm should review their experimental designs carefully regarding potential response strategies. Once these are taken into account, the paradigm can be used to study lexical-semantic encoding in different populations of healthy and also impaired speakers.

## Introduction

Retrieving words from the mental lexicon requires that speakers activate potential lexical candidates in their mental lexicon and select one of them for lexicalization. How a target entry is selected is a matter of ongoing debate. According to Roelofs (1992) and Levelt et al. (1999) co-activated lexical entries compete for selection (see also Howard et al., 2006). For selection to occur, the activation of one lexical entry needs to exceed the summed activation of all its competitors. By contrast, Dell (1986) and Oppenheim et al. (2010; see also Navarrete et al., 2014) assume that lexical selection is not by competition but is enforced at some point, targeting the most activated lexical entry at the time of selection.

**FIGURE 1:**
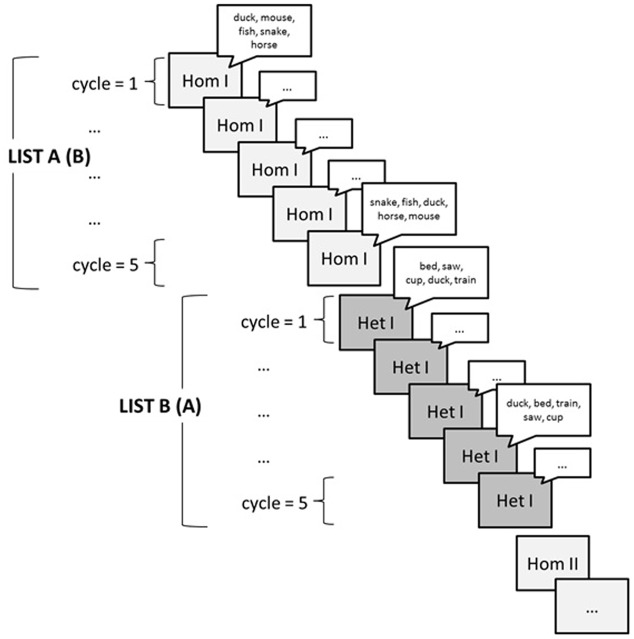
**Schematic representation of a standard blocked-cyclic naming paradigm: Small object sets are combined to lists of objects in a cyclic fashion**. In testing, the lists are administered in alternation (as shown), in random order, or in a blocked fashion.

Of particular relevance for testing predictions from the different theories of lexical selection are semantic interference effects, which arise when participants name objects in the presence of semantically related words. In the experimental paradigm the present review is concerned with – blocked-cyclic naming – participants repeatedly name small sets of objects from the same semantic category (homogeneous context) or from different semantic categories (heterogeneous context). The objects in the sets are compiled to longer lists in a cyclic fashion (Belke et al., 2005b), that is, all objects of a set are presented repeatedly in varying orders, such that all members of a set are named once before a new presentation cycle is initiated (see **Figure 1**). Participants’ response times are substantially slower in homogeneous than in heterogeneous contexts. The proponents of the two modeling traditions introduced above have different takes on this finding, as I will review shortly. In this paper, I argue that key findings from the blocked-cyclic naming paradigm can only be accounted for when response strategies are taken into account.^1^

## Blocked-Cyclic Naming: Central Findings and Implications

In standard blocked-cyclic naming experiments (**Figure 1**), the semantic interference effect emerges from cycle two onward only. In cycle 1, there is either no context effect or a semantic facilitation effect. Ever since this pattern of results was first documented (Belke et al., 2003, 2005b; Damian and Als, 2005), it has been replicated reliably in virtually all studies using the standard blocked-cyclic naming paradigm. Using variants of the blocked-cyclic paradigm, Damian and Als (2005) and Belke (2013) demonstrated that the interference effect seen is long-lasting – it persists even when participants name unrelated objects in between the related ones (such as body parts mixed with unrelated items: *finger*, chicken, *ear*, bed, boxer, *nose*, *mouth*, crown, …; see also Navarrete et al., 2012). This longevity of the effect is in keeping with long-lasting semantic context effects observed in related naming paradigms (e.g., Howard et al., 2006).

To account for these findings, models of lexical selection have been augmented by incremental learning mechanisms that modify the strength of the links between conceptual representations and their associated lexical representations, inducing long-lasting changes to the dynamics of spreading activation in the system of lexical-semantic representations (Howard et al., 2006; Oppenheim et al., 2010; see also Belke, 2013):

Proponents of models of lexical selection by competition argue that the lexical-semantic representation of an object that has been named is strengthened by means of an incremental learning mechanism operating at the interface of the conceptual and the lexical-level representations (Damian and Als, 2005; Howard et al., 2006) or at the conceptual level (Belke, 2013). In the heterogeneous context, this causes the targets to become increasingly easily accessible over cycles, as, within their respective categories, they are the only exemplars whose representations are strengthened in this way. In the homogeneous context, by contrast, the incremental learning mechanism renders the retrieval of any given object name increasingly hard. This is because in each cycle the representations of several exemplars of the same semantic category as the target are strengthened and subsequently present stronger competitors in the retrieval of the name of another target.

In models of lexical selection without competition, the learning mechanism not only strengthens the links between the target’s semantic features and its lexical representation but it also weakens the links between the semantic features and the lexical representations of all representations that were co-activated in the naming process but were not selected (Oppenheim et al., 2010). In a homogeneous context, a lexical entry features as the target once per cycle while otherwise being a co-activated but non-selected category co-ordinate. As a result, its representations are weakened more often than they are strengthened and it has an increasingly hard time to accumulate sufficient activation for selection. In heterogeneous contexts, by contrast, a given set member is never co-activated when another set member is being named, so that the repetition priming effect induced by the strengthening of the target’s representations can take its full effect.

Functionally, both accounts of semantic context effects in blocked-cyclic naming involve a competitive component, albeit at different stages of processing: While in the models put forward by Levelt et al. (1999) and Howard et al. (2006) lexical selection is a competitive process, the model by Oppenheim et al. (2010) incorporates a competitive component in the incremental learning mechanism, i.e., at a post-selection stage of processing. Navarrete et al. (2012, 2014) have pointed out that the difference in localizing competition effects in the system has substantial implications on how the two approaches account for semantic context effects. Most importantly, they deem different aspects of the data gained from blocked semantic context manipulations as relevant with respect to the investigation of lexicalization processes: Based on the model put forward by Oppenheim et al. (2010), Navarrete et al. (2012, 2014) argued that what appears to be a semantic interference effect is, in fact, the result of reduced repetition priming in the homogeneous as compared to the heterogeneous context. This is because in the homogeneous context, the strengthening that a set member experiences upon having been named (repetition priming) is counteracted by the weakening incurred by other set members being named in the same cycle. Consequently, the repetition priming effect is reduced, creating the significant difference between the homogeneous and heterogeneous contexts seen in the data. Navarrete et al. argued that, by implication, the effects seen as of cycle 2 onward bear no immediate relevance to theories of lexical access; instead, the effect seen in the first cycle is the most relevant data point. With this interpretation of the data, Navarrete et al. (2014) take context effects to be driven purely from the bottom up, constituting the net result of semantic priming, incremental learning or repetition priming, and lexical selection without competition.

Proponents of models of lexical selection by competition have argued that there is typically no effect or facilitation in the first cycle, because semantic priming effects at the conceptual level cancel out or outweigh the lexical interference effect (Damian and Als, 2005; Abdel Rahman and Melinger, 2007; Belke, 2013). As of cycle 2, lexical interference outweighs semantic facilitation, causing a net interference effect. Repetition priming is not seen as a key factor; it is taken to be of similar magnitude in homogeneous and heterogeneous contexts. The semantic facilitation effect in cycle 1 is typically accounted for by short-lived semantic priming effects (Wheeldon and Monsell, 1994) that are overridden by longer-term semantic interference induced by the incremental learning mechanism by the second or third cycle (Damian and Als, 2005; Belke, 2013). Note that this longer-term semantic interference effect may have a dual, conceptual and lexical, locus, with semantic facilitation accumulating at the conceptual level, causing cumulative interference at the lexical level (Abdel Rahman and Melinger, 2007; Belke, 2013).

Both types of account reviewed so far take the semantic facilitation effect to be the result of processes inherent to the lexicalisation process. However, over the years, both proponents of theories of lexical selection and proponents of theories of lexical selection without competition have suggested that the facilitation effect seen in cycle 1 is strategic (Damian and Als, 2005; Oppenheim et al., 2010). In that case, it would have little bearing on theories of lexical selection.

## Evidence for Strategic Facilitation in Cycle 1

In the following, I demonstrate that a seemingly minor design feature – the way in which homogeneous and heterogeneous lists are administered in a blocked-cyclic naming task – is key to the emergence or absence of the semantic facilitation effect in cycle 1. A list is the sequence of objects resulting from cycling through a set a number of times, say five times (**Figure 1**). Studies using the blocked-cyclic naming paradigm differ with respect to the presentation of lists to participants. While some have used a random (Schnur et al., 2006) or an alternating order (e.g., Belke et al., 2005b), others feature a blocked presentation of lists by contexts (e.g., Abdel Rahman and Melinger, 2007; Crowther and Martin, 2014; Marful et al., 2014).

I will first look at the results of studies that have blocked lists by context. This order is schematically represented as AAAABBBB and AABBBBAA, with A being a homogeneous list and B being a heterogeneous list for half of the participants and vice versa for the others. **Table 1** (top) presents the data for 10 such data sets from healthy speakers. It provides the magnitude of the effect in cycle 1 in ms, the results of paired *t*-tests of this effect by participants/items, estimates of the effect size (*Hedges’ g*_av_, *Cohen’s d*_z_; Lakens, 2013) and the statistical significance of the interaction of context and presentation cycle when all cycles are included and when the first cycle is excluded. Looking at the effects reported for cycle 1, we see consistent and significant facilitation with small to medium-sized effects in seven of the 10 data sets. Abdel Rahman and Melinger (2011, Experiments 1 and 2) found no facilitation in cycle 1. In Navarrete et al. (2012, semantically far object sets), the effect was facilitatory descriptively but was not statistically significant, possibly due to a lack of statistical power (*N* = 12). In all data sets, there is a significant context x cycle interaction when all cycles are included.

**Table 1:** Blocked-cyclic naming experiments featuring (a) blocks of homogeneous and heterogeneous lists or (b) homogeneous and heterogeneous lists in alternation: Magnitude, statistical significance (by participants/items, where applicable), and effect size (Hedges’ *g*_av_ and Cohen’s *d*_z_) of the context effect in cycle 1, and interactions of context and presentation cycle when all cycles are included and when the first one is excluded.

			Blocked presentation of homogeneous and heterogeneous lists

	**List presentation**	**# Ppts**	**# Cycles**	**Effect in 1st cycle (paired *t*-tests)**	***g*_av_^a^**	***d*_z_^a^**	**Interaction all cycles**	**Interaction, excldg. 1st cycle**
Abdel Rahman and Melinger, 2007 (Experiment 1, categorically homogeneous sets only)	AAAABBBB	30	4	-31 ms	^∗^/^∗∗∗^	0.364	0.438	^∗∗∗^/^∗∗∗^	n.s./n.s.
Abdel Rahman and Melinger, 2007 (Experiment 2, categorically homogeneous sets only)	AAAABBBB	30	6	-36 ms	^∗^/^∗∗∗^	0.438	0.402	^∗∗∗^/^∗∗∗^	^∗^/n.s.
Abdel Rahman and Melinger, 2011^b^ (Experiment 1, categorically homogeneous sets only)	AAAABBBB	24	6	-1 ms	n.s./n.s.	0.019	0.024	^∗^/^∗∗∗^	n.s./n.s.
Abdel Rahman and Melinger, 2011^b^ (Experiment 2, categorically homogeneous sets only)	AAAABBBB	24	6	-10 ms	n.s./n.s.	0.088	0.095	^∗^/^∗∗∗^	n.s./n.s.
Marful et al., 2014 (Experiment 1, object naming)	AAAABBBB	24	4	-30 ms^c^	^∗∗∗^	0.291^c^	–	^∗∗∗^	n.s.
Navarrete et al., 2012 (Experiment 1)	AABBBBAA	12	4	-15 ms	^∗^/^∗∗^	0.220	0.667	^∗∗∗^/^∗∗∗^	–
Navarrete et al., 2012 (Experiment 2) semantically close	AABBBBAA	12	4	-21 ms	^∗^/^∗∗^	0.403	0.722	^∗^/^∗^	–
Navarrete et al., 2012 (Experiment 2) semantically far	AABBBBAA	12	4	-13 ms	n.s./n.s.	0.230	0.436		
Janssen et al., 2015^d^	AABBBBAA	26	4	-19 ms	^∗^	0.120	–	^∗∗∗^	n.s.
Crowther and Martin, 2014	AABBBBAA	82	4	-22 ms	^∗∗∗^	0.232^e^	0.632	^∗∗∗^/^∗∗∗^	–
		Average effect		**-22 ms^f^**		**0.240**	**0.427**	

			**Alternating presentation of homogeneous and heterogeneous lists**

Belke et al., 2005b, Experiment 1	ABABABAB	16	8	9 ms	n.s./n.s.	0.127	0.348	^†^/n.s.	n.s./n.s.
Belke et al., 2005b, Experiment 3, consistent sets^g^	ABABABAB	24	8	-3 ms	n.s./n.s.	0.033	0.062	^∗∗∗^/^†^	n.s./n.s.
Belke et al., 2005a (Experiment 1, early acquired names)	ABABABAB	20	6	12 ms	n.s./n.s.	0.200	0.340	n.s./n.s.	n.s./n.s.
Belke et al., 2005a (Experiment 1, late acquired names)	ABABABAB	20	6	-12 ms	n.s./n.s.	0.170	0.306	^∗∗^/^∗∗∗^	n.s./n.s.
Belke and Meyer, 2007 (Experiment 1, young speakers)	ABABABAB	16	8	-11 ms	n.s./n.s.	0.112	0.268	^∗∗^/^∗∗∗^	n.s./n.s.
Belke and Meyer, 2007 (Experiment 1, older speakers, 52–88 years.)	ABABABAB	16	8	-9 ms	n.s./n.s.	0.084	0.118	n.s./^†^	n.s./n.s.
Belke, 2008 (Experiment 1, no WM-load)	ABABABAB	20	5	3 ms	n.s./n.s.	0.062	0.123	^∗∗∗^/^∗∗∗^	^∗^/^∗∗^
Damian and Als, 2005 (Experiment 4A)^h^	ABABABAB	16	4	-4 ms	n.s./n.s.	0.056	0.149	^∗^/^∗^	n.s./n.s.
		Average effect		**-2 ms^f^**		**0.106**	**0.214**		

^†^*p < 0.08; ^∗^*p* < 0.05; ^∗∗^*p* < 0.01; ^∗∗∗^*p* < 0.001. ^a^*Hedges’ g*_av_ = (M_hom_*–M*_het_*)/√[(SD*^2^_hom_*+SD*^2^_het_*)/2)]*, *Cohen’s d*_z_* = t/√n*. ^b^Results of paired t-tests as reported by Abdel Rahman (personal communication). ^c^Effect in first cycle as reported by Marful (personal communication). ^d^Effect size and results for interactions (interaction all cycles; interaction, excluding 1st cycle) as reported by Janssen (personal communication). ^e^Effect size estimate based on standard deviations provided by Crowther and Martin (personal communication). ^f^Weighted average, taking into account the number of participants per experiment. ^g^For the present analyses, the data were collapsed across the two participant groups. ^h^Results of paired t-tests as reported by Damian (personal communication)*.

These results differ markedly from those seen in experiments featuring an alternating order of homogeneous and heterogeneous lists (ABABABAB). As the paired *t*-tests show, there is no significant context effect in cycle 1 in any of the 8 data sets from ABABABAB-experiments. The effect ranges from -12 to 12 ms, averaging -2 ms. The absence of the effect in cycle 1 typically yields a significant context x cycle interaction, except, of course, in those cases when the participants’ naming latencies are slower in the homogeneous than in the heterogeneous context in cycle 1 (Belke et al., 2005a,b, Experiment 1, early acquired object names; but see Belke and Meyer, 2007, older speakers).

Note that, on average, the number of participants in the experiments with alternating list orders was smaller (*N* = 19) than in experiments with blocked list orders (*N* = 28), so the alternating list experiments might lack statistical power. To test this, I first computed the effect size of the 22-ms facilitation effect in the blocked design using G^∗^Power (Faul et al., 2007), *d*_z_ = 0.4551.^2^ Compared to the experimental power in the blocked designs (1-β = 0.758 for a one-sided *t*-test, α = 0.05), experimental power did not drop dramatically for the smaller average number of participants in the alternating designs (*N* = 19; 1-β = 0.633).

Clearly, designs that highlight implicitly the semantic relatedness of the items in the homogeneous lists by blocking by list context typically cause facilitation in cycle 1, whereas designs that do so less apparently are associated with no effect in cycle 1. This pattern cannot be accounted for in a straightforward fashion by a theory that takes semantic facilitation and repetition priming as the only driving forces of the effects seen in cycle 1 and thereafter, as advocated by Navarrete et al. (2014). Such a theory would predict that the amount of facilitation is identical regardless of the order in which the homogeneous and heterogeneous lists are administered. The same holds for theories that take the early facilitation effect to be a trade-off between lexical interference and short-lived semantic priming effects at the conceptual level (Damian and Als, 2005; Abdel Rahman and Melinger, 2007; Belke, 2008). Instead, it seems more appropriate to assume that at least some of the facilitation seen in the studies featuring a blocked administration of homogeneous and heterogeneous naming lists is strategic, with participants putting their awareness of the current semantic category to use in order to facilitate lexical retrieval. Alternatively, they may use the semantic information in order to enhance top-down the visual recognition of objects that are difficult to recognize (Abdel Rahman and Melinger, 2007; Aristei et al., 2011).

## Non-Cumulative Interference in Blocked-Cyclic Naming

It is noteworthy that both accounts of lexical retrieval reviewed in the introduction make the strong prediction that the interference effect increases from cycle to cycle. This is because with each new homogeneous cycle, lexical selection is getting harder while it is getting easier across heterogeneous contexts. However, when tracing the effect across cycles, most studies have found that, in healthy speakers, the effect emerges in cycle 2 and does not increase thereafter (e.g., Belke et al., 2005b; Damian and Als, 2005), much unlike the effect seen in the continuous paradigm (see Belke and Stielow, 2013, for an overview). Belke (2008, 2013; see also Belke and Stielow, 2013) has argued that this is because the paradigm allows participants to encode, during the first cycle, the objects featuring in each set and to subsequently bias, top-down, the representations of the objects in the set over other lexical representations. In the heterogeneous context, this induces a genuine processing advantage, as the competition among joint category members is biased toward a single exemplar per category. In the homogeneous context, by contrast, the bias is less effective: While it also allows speakers to bias the competition toward the set members, it does not alleviate the competition among the set members, which are typically all category associates. By implication, there is a consistent processing advantage of heterogeneous over homogeneous items, causing the semantic interference effect observed in most experiments. Cumulative semantic interference induced by incremental learning is counteracted by the top-down bias, explaining why the interference effect in blocked naming does not accumulate.

There are now several lines of research that support this account. Belke (2008) has shown that when speakers’ working memory is loaded by a concurrent digit retention task, they display bigger context effects, arguably because they are less efficient at biasing the set members top-down. Likewise, patients suffering from neurological damage to these left frontal areas show cumulative semantic context effects in blocked-cyclic naming, arguably because their ability to bias relevant set members top-down is impaired (see Belke and Stielow, 2013; but see Ries et al., 2015). So far, Howard et al. (2006), Oppenheim et al. (2010), and Navarrete et al. (2014) have not addressed how their accounts would explain these findings for healthy and impaired speakers and for blocked as compared to continuous manipulations of semantic context.

## Conclusion

The findings I have reviewed in this paper suggest that response strategies play an important role in the emergence of semantic facilitation and interference in blocked-cyclic naming. Facilitation in cycle 1 is observed most reliably when the homogeneous and heterogeneous lists are presented in a blocked fashion, as compared to an alternating or random presentation. I argue that this is the case because a blocked presentation makes participants more aware of the semantic relatedness in a large part of the sets, allowing them to identify the semantic category of the items in the homogeneous sets and to use this knowledge strategically. Models attempting to explain the effects with reference to mechanisms inherent to the lexicalisation process alone cannot accommodate such effects of the experimental design on the magnitude of the facilitation effects.

Similarly, the finding that semantic context effects typically do not cumulate over cycles in healthy speakers can be readily explained by participants biasing the members of the naming sets top-down. Models that do not incorporate this response strategy make the incorrect prediction that semantic context effects invariably cumulate over cycles.

The findings reviewed in this article demonstrate that it is not the case that the data from cycle 2 onward are theoretically irrelevant, as Navarrete et al. (2014) have claimed. Instead, the data from all cycles are relevant and useful for studying lexical-semantic encoding. The blocked(-cyclic) naming paradigm is a rather easy naming task, as it involves a limited number of stimuli and numerous repetitions. This renders it ideally suited for studying lexical-semantic encoding in different populations of healthy and impaired speakers, provided that users of the paradigm have a clear understanding of the way it works. With a full understanding of the influences impacting on semantic context effects, it is possible to apply the paradigm to exploring the structure of the mental lexicon across the lifespan and across populations. Furthermore, the paradigm is most suited for studying the effects of neuropsychological disorders of language and its interplay with executive functions (see Belke and Stielow, 2013 for an overview).

## Author Contributions

EB carried out the review reported in this paper and wrote the paper.

## Conflict of Interest Statement

The author declares that the research was conducted in the absence of any commercial or financial relationships that could be construed as a potential conflict of interest.

## Footnotes

1 I consider behavioral findings only, as the relevant electrophysiological studies have either used non-standard variants of the blocked-cyclic paradigm (Aristei et al., 2011) or yielded inconsistent electrophysiological signatures of context effects (Janssen et al., 2011, 2015; Llorens et al., 2014). These inconsistencies might be accounted for by differences in administering the experiments and analyzing the data; however, space limitations do not allow for a detailed discussion of such differences in this review.

2 To estimate the correlation between the naming latencies obtained in homogeneous and heterogeneous contexts in cycle 1, I established the average correlation in the data sets I co-authored (**Table 1**), *r* = 0.875.
